# Identification of Tumor-Specific MRI Biomarkers Using Machine Learning (ML)

**DOI:** 10.3390/diagnostics11050742

**Published:** 2021-04-21

**Authors:** Rima Hajjo, Dima A. Sabbah, Sanaa K. Bardaweel, Alexander Tropsha

**Affiliations:** 1 Department of Pharmacy, Faculty of Pharmacy, Al-Zaytoonah University of Jordan, P.O. Box 130, Amman 11733, Jordan; dima.sabbah@zuj.edu.jo; 2 Laboratory for Molecular Modeling, Division of Chemical Biology and Medicinal Chemistry, Eshelman School of Pharmacy, The University of North Carlina at Chapel Hill, Chapel Hill, NC 27599, USA; alex_tropsha@unc.edu; 3 National Center for Epidemics and Communicable Disease Control, Amman 11118, Jordan; 4 Department of Pharmaceutical Sciences, School of Pharmacy, University of Jordan, Amman 11942, Jordan; s.bardaweel@ju.edu.jo

**Keywords:** biomarkers, imaging, machine learning, MRI, oncology

## Abstract

The identification of reliable and non-invasive oncology biomarkers remains a main priority in healthcare. There are only a few biomarkers that have been approved as diagnostic for cancer. The most frequently used cancer biomarkers are derived from either biological materials or imaging data. Most cancer biomarkers suffer from a lack of high specificity. However, the latest advancements in machine learning (ML) and artificial intelligence (AI) have enabled the identification of highly predictive, disease-specific biomarkers. Such biomarkers can be used to diagnose cancer patients, to predict cancer prognosis, or even to predict treatment efficacy. Herein, we provide a summary of the current status of developing and applying Magnetic resonance imaging (MRI) biomarkers in cancer care. We focus on all aspects of MRI biomarkers, starting from MRI data collection, preprocessing and machine learning methods, and ending with summarizing the types of existing biomarkers and their clinical applications in different cancer types.

## 1. Introduction

Imaging is routinely used for cancer diagnosis and staging, for monitoring treatment efficacy, for detecting disease recurrence, or generally for cancer surveillance [1,2,3,4]. Understanding the anatomical and physiological aspects of medical images allows experts to distinguish aberrant from normal appearance [5]. Advances in analytical methods and the application of machine learning methods enabled the use of medical images as biomarkers that can potentially optimize cancer care and improve clinical outcome [5]. The imaging biomarkers that are currently, and successfully, used for clinical diagnosis have attracted many researchers’ attention as described in multiple publications [1,5,6,7,8,9,10,11,12,13,14,15,16,17,18].

Magnetic resonance imaging (MRI) is a diagnostic imaging technique that applies strong magnetic and radio waves to generate high quality MRI scans of body organs facilitating the diagnosis of tumors and other conditions such as brain and spinal cord diseases. Currently, MRI is one of the of the big data producers in biomedicine, and is being exploited as important generator of cancer biomarkers. In essence, a biomarker is a characteristic that is measured as an indicator of a biological condition of interest (i.e., normal biological processes, pathogenic processes, or responses to a therapeutic intervention) [19,20]. The process of biomarker prioritization starts with a theory and ends with biomarker validation in an experimental setting. However, the current dogmas in biomedicine may hinder the process of unbiased hypothesis generation due to the complexity of cancer phenotypes and patient attributes, which makes it harder for human experts and physicians to comprehend all the details in MRI scans [21]. This led to the rise MRI biomarkers, identified by ML, that could capture disease characteristics with high accuracy, efficiency, reproducibility and interpretability [5,22].

## 2. Imaging Biomarkers

Biomarker stands for biological marker and it is defined by the U.S. Food and Drug Administration (FDA) as “a defined characteristic that is measured as an indicator of normal biological processes, pathogenic processes, or responses to an exposure or intervention, including therapeutic interventions” [23]. Biomarkers can measure anatomical, histological, physiological, molecular, and radiographic characteristics. Imaging biomarkers are convenient and reliable [5]. In oncology, they represent comprehensive cancer features such as apoptosis, angiogenesis, growth, metabolism, invasion, metastasis, and selective target interaction [24]. Cancer imaging biomarkers are widely used for cancer identification, for the prediction of disease outcome, and for monitoring treatment responses [5]. Examples of imaging biomarkers include Tumor, Node, Metastasis (TNM) reflecting a staging system (i.e., a prognostic biomarker) and Response Evaluation Criteria in Solid Tumors (RECIST) which can be applied as a response biomarker [1]. Confirmed imaging biomarkers are used to support decision-making in clinical practice. The necessity for quantitative evaluation in diagnosis must be validated [5]. Quantitative approach is profound and exhaustive due to technology and apparatus differences as well as quantitative development that influences the extracted data [5]. The well-established QA and QC protocols are perquisite to validate and approve the reliability of medical assessment along with endeavor made by research, radiological, and medical institution [5]. In addition, significant factors should be considered such as isolating normal healthy from ailment tissues to achieve better diagnosis [5]. Table 1 provides a summary of the various types of imaging biomarkers used in cancer besides MRI.

## 3. MRI Biomarkers

MRI can be exploited to extract numerous variables according to diverse inherent tissue properties such as proton density, diffusion, and T1-and T2 relaxation times [1]. In addition, MRI can probe the alterations in parameters due to the association of macromolecules and contrast agents [5]. For example, the apparent diffusion coefficient (ADC) is an extensively used criterion in cancer identification [16,62], diagnosis, and treatment assessment [63,64]. However, post-processing tools to derive absolute quantitation are widely disputed [65,66,67], although the protocol itself is versatile and reliable for cancer detection [68]. Quantification of T1 relaxation has an impact on cardiovascular MRI rather than depending on image contrast [69]. T1 values are significant in differentiating cardiac inflammation [70], multiple sclerosis [71,72], liver fat and iron concentration [73,74], and endocrine glands [75].

Quantitative chemical exchange saturation transfer (CEST) imaging is promising in evaluating brain ischemic disease [76], osteoarthritis [77], lymphedema [78], cancer pH and metabolomics [79]. Furthermore, MRI offers beneficial effects such as optimum images distinction, superior resolution, providing many contrasts per each testing; probing histological features (oxygenation, perfusion, and angiogenesis) [1].

Distinctive MRI biomarkers have been assigned in cancer diagnosis [1] including Breast Imaging Reporting and Data System (BI-RADS) [2], Liver Imaging Reporting and Data System (LI-RADS) [80,81], Prostate Imaging Reporting and Data System (PI-RADS) [4], TNM, and RECIST [1]. Quantitative biomarkers have been employed in clinical research studies such as initial area under the gadolinium curve (iAUGC) or transfer constant (K^trans^) from dynamic gadolinium enhanced (DGE) imaging and apparent diffusion coefficient (ADC) [1]. Morphological-based cancer biomarkers use many contrasts and moderate to high spatial resolution of MRI [1,82,83,84]. T1-weighted and T2-weighted imaging are utilized in cancer profiling [1].

## 4. MRI Data Preprocessing

Applying machine learning directly on raw MRI scans often yields poor results due to noise and information redundancy. Furthermore, machines read and store images in the form of number matrices. Raw MRI data are transformed into numerical features that can be processed by machines while preserving the information in the original data set.

## 5. Machine Learning for MRI Data

**Figure 1:**
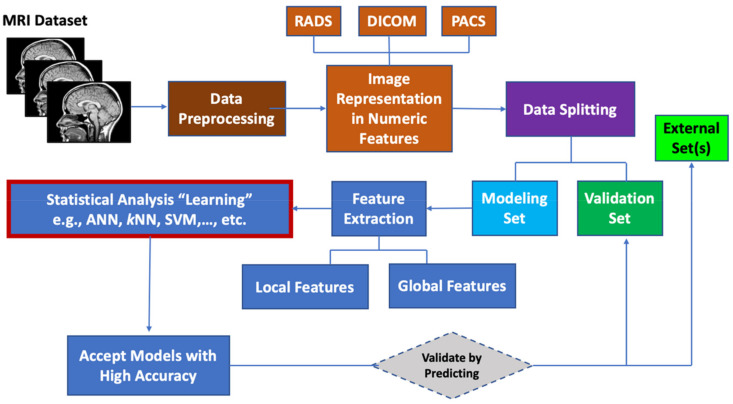
Workflow for prioritizing ML MRI biomarkers.

Machine learning (ML) algorithms are becoming useful components of computer-aided disease diagnosis and decision support systems. Computers seem to be able to recognize patterns that humans cannot perceive. Hence, ML provides a tool to analyze and utilize a massive amount of data more efficiently than the conventional analysis carried by human. This realization has led to heightened interest in ML and AI applications to medical images. Recently, employing ML in analyzing big data resulting from medical images, including MRI data, have been useful in obtaining significant clinical information that can aid physicians in making important decisions regarding clinical diagnosis, clinical prognosis, or treatment outcome [55,85,86]. ML can be used also to prioritize MRI biomarkers. The workflow for prioritizing MRI biomarkers using ML is summarized in Figure 1.

### 5.1. Image Representation by Numeric Features

The success of machine learning relies on data representation [87]. MRI images are represented in terms of features which are numeric values that can be processed by machines. These numeric values could be actual pixel values, edge strengths, variation in pixel values in a specific region of the MRI image, or any other value [88]. Non-image features can be also used in the machine learning process and may include age of the patients, the outcome of the laboratory test, sex, and other available patient or laboratory attributes. Features can be combined to form a feature vector which is also called the input vector [88].

### 5.2. Feature Extraction

Feature extraction, also known as feature engineering, is the process of identifying the most distinguishing characteristics in imaging signals that characterize MRI images and describe their behavior, allowing machine learning methods to process imaging data and learn from these data. Features can be referred to as descriptors. Feature extraction can be accomplished either manually or automatically.

Image features are usually classified into two main groups: global and local. Global features are generated as a d-dimensional feature vector which represents a specific pattern [89]. Global features usually describe the color, shape, and texture, and are commonly applied in content-based image retrieval (CBIR) systems [90,91,92,93,94,95,96]. Local features refer to certain patterns or specific structures on images that distinguish them from their surroundings. Examples of local features include blobs, corners, and edge pixels [97].

### 5.3. Data Set Division for Model Building, Model Tuning and External Validation

Many machine learning methods require model training with previously labeled MRI data. For generating these models, the data is divided into three sets: training set, test set and an external validation set that is not used in any way for model building. The modeling set (that remains after splitting out the validation set) is split additionally into training and testing (or tuning) sets. If models fail to predict the external validation set, such models are discarded and not used to make predictions. Additionally, other independent validation sets may become available after the completion of the modeling studies, and then can be used as additional validation sets. We have shown earlier that training-set-only modeling is not sufficient to obtain reliable models that are externally predictive [98,99]. Models that are highly predictive on training and testing data should be retained for the majority voting on external validation sets. Finally, only those models shown to be highly predictive on both testing and external validation sets are used as robust classifiers for MRI imaging data.

### 5.4. Machine Learning Algorithms

Machine learning algorithms generate models that can classify MRI images into malignant and benign based on extracted local and global image features. The generated ML model is a mathematical model that can predict outcome by generalizing their learned experience on training set data, to deliver a correct prediction of new MRI images unseen by the developed models. The learning exercise can be supervised, semi-supervised or unsupervised. However, for imaging data we rely heavily on supervised methods that can be applied to class-labeled data.

**Figure 2:**
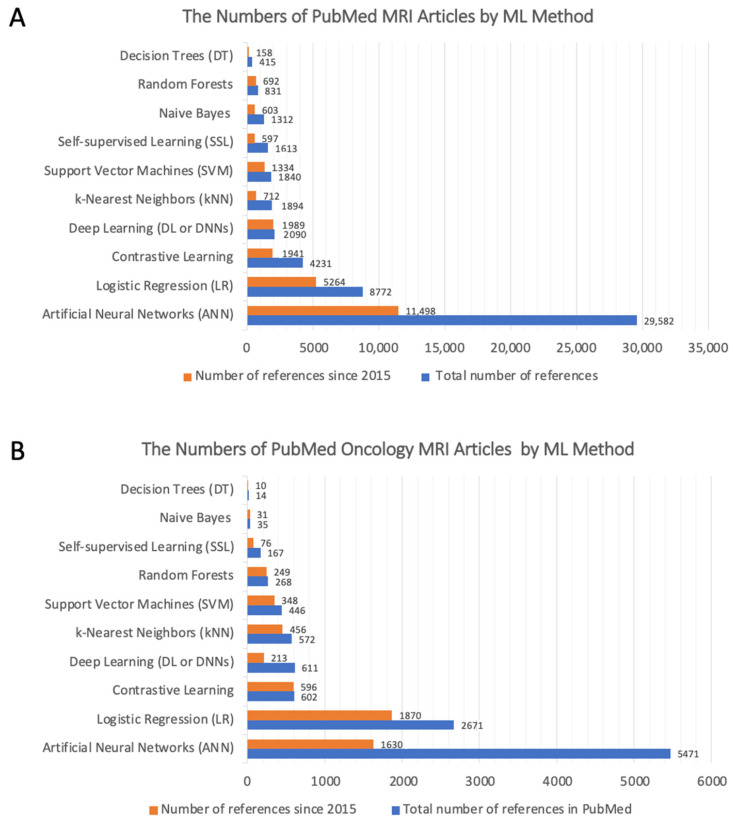
Column chart showing the number of MRI articles based on the ML method used. (**A**) The total number of PubMed MRI articles based on the applied ML method. (**B**) The total number of PubMed Oncology MRI articles based on the applied ML method.

There are three main challenges to applying machine learning in medical imaging for cancer diagnosis: classification, localization, and segmentation. We need ML methods to overcome all these challenges. Herein, we review the most popular ML algorithms applied for MRI biomarkers, and results summarized in Figure 2. We also discuss advantages and disadvantages of each method (Table 2).

#### 5.4.1. Artificial Neural Networks

Learning with artificial neural networks (ANNs) is one of the most famous machine learning methods that was introduced in the 1950s, and is being employed for classifying MRI data [103]. The generated neural network consists of a number of connected computational units, called neurons which are arranged in layers. There is an input layer that allows input data to enter the network, followed by hidden layer or layers transforming the data as it flows through, before ending at an output layer that produces the neural network’s predictions. The network is trained to generate correct predictions by identifying predictive features in a set of labeled training data, fed through the network while the outputs are compared with the actual labels by an objective function [103]. Furthermore, message passing neural network (MPNN) has distinguished morphological aspects in benign and malignant cancers [104]. Diverse morphological features have been recognized including elliptic-normalized circumference (ENC), elliptic-normalized circumference (ENC), long axis to short axis (L:S), abrasions’ sizes, and lobulation index (LI) [67].Further features have been distinguishes such as branch form, nodule brightness, lobulations’ numbers, and ellipsoid features [105].

The ANN method is composed of three learning schemas: (1) the error function which measures how good or bad an output is for a given input, (2) the search function which defines the direction and magnitude of the change required to reduce the error function, and (3) the update function which defines how the weights of the network are updated on the basis of the search function values [88]. This is an iterative process which keeps adjusting the weights until there is no additional improvement. ANN models are very flexible, capable of solving complex problems, but they are difficult to understand and very computationally expensive to train [103].

#### 5.4.2. Logistic Regression (LR)

Logistic regression is a statistical model that uses a logistic function to model binary dependent variable (y) in MRI classification data. It models the probability of that the MRI is for tumor versus normal tissue by using a linear model to predict the log-odds that that y = 1; and then uses the logistic/inverse logit function to convert the log-odds values into probabilities [106]. However, LR models tend to overfit high-dimensional data. Therefore, regularization methods are often used to prevent overfitting to training set data. Regularization is achieved by using a model that tries to fit the training data well, while at the same time trying not to use regression weights that are too large [107]. The most common approaches are L1 regularization, which tries to keep the total absolute values of the regression weights low, and L2 or ridge regularization, which tries to keep the total squared values of the regression weights low.

#### 5.4.3. Contrastive Learning

Contrastive learning is a ML technique that can learn the general features of a dataset (i.e., the MRI dataset) without labels, by teaching the model which data points are similar or different. This can be formulated as a dictionary look-up problem. This algorithm is considered a particular variant of self-supervised learning (SSL) that is particularly useful for learning image-level representations [108]. One of the advantages of this method is that it can be applied for semi-supervised learning problems when clinical annotations are missing from MRI data. This method permits the use of both labeled and unlabeled data to optimize the performance and learning capacity of the classification model. A method that has gained popularity in the literature recently is the unsupervised pre-train, supervised fine-tune, knowledge distillation paradigm [109].

#### 5.4.4. Deep Learning

Deep learning which is also known as deep neural network (DNNs), or deep structured learning, is a machine learning method based on artificial neural networks which allows computational models that are composed of multiple processing layers (typically more than 20 layers) to learn representations of data with multiple levels of abstraction [110]. In deep learning, the algorithm learns useful representations and features automatically, directly from the raw imaging data. By far the most common models in deep learning are various variants of ANNs, but there are others as well [103]. Deep learning methods primarily differ from “classical” machine learning approaches by focusing on feature learning, i.e., automatically learning representations of data [103]. In medical imaging the interest in deep learning is mostly triggered by convolutional neural networks (CNNs) [111]. Features are automatically deduced and optimally tuned for the desired outcome. Deep learning protocols have been applied in cancer prognosis such as melanoma, breast cancer, brain tumor, and nasopharyngeal carcinoma [112,113,114,115].

However, models based on deep learning are often vulnerable to the domain shift problem, which may occur when image acquisition settings or imaging modalities are varied [108]. Further, uncertainty quantification and interpretability may additionally be required in such systems before they can be used in practice. Many strategies have been used to improve the performance of DNNs including contrastive learning, self-organized learning, and others. Recently, FocalNet has become one of the preferred iterative information extraction algorithms to be used with DNNs. This algorithm uses the concept of foveal attention to post-process the outputs of deep learning by performing variable sampling of the input/feature space [116]. FocalNet is integrated into an existing task-driven deep learning model without modifying the weights of the network, and layers for performing foveation are automatically selected using a data-driven approach [116].

#### 5.4.5. *k*-Nearest Neighbors (*k*NN)

The *k*NN method is based on the *k* nearest neighbors’ principle and the variable selection procedure for feature selection reviewed elsewhere [98,117]. The procedure starts with the random selection of a predefined number of features from all selected features. The generated model can then classify an input vector of a new MRI image (i.e., a collection of MRI image features) by assigning it to the most similar class based on the number of neighbors (i.e., *k*) with known class labels, that vote on which class the input object belongs to. The predicted class will be the result of majority voting of all *k* nearest neighbors.

#### 5.4.6. Support Vector Machines (SVM)

Support-vector machines (SVM) are supervised learning models that apply associated learning algorithms for data analysis; they can be used for classification and regression tasks [118,119]. They are named support vector machines because they transform input data in a way that produces the widest plane, or support vector, of separation between the two classes. SVMs gained popularity because they can classify data that are not linearly separable.

#### 5.4.7. Random Forests

The random forests algorithm is a ML technique that uses an ensemble model to make predictions [120]. It essentially uses a bundle of decision trees to make a classification decision. Since, ensemble models implement the results from many different models to calculate a response or to assign a class, they perform better than individual models, and increasingly being used for image classification [98,121]. Random forests algorithm can handle big data, can estimate missing data without compromising accuracy, less prone to overfitting than decision trees, it works well for unbalanced datasets and for classification problems. However, it works like a black box with minimum control on what the model does, and models are difficult to interpret.

#### 5.4.8. Self-Supervised Learning

Self-supervised learning (SSL) provides a strategy to pre-train a neural network with unlabeled data, followed by fine-tuning for a downstream task with limited annotations, e.g., such as in clinical data, to yield high predictive performance [109,122]. However, extensive validation of the automated algorithms is essential before they can be used in critical decision making in healthcare. One of the self-supervised learning methods that showed improved performance on deep learning models applied a strategy based on ‘context restoration’ to handle unlabeled imaging data [122]. The context restoration strategy is characterized by: (1) its ability to learn semantic image features; (2) it uses the learned image features for subsequent image analysis tasks; and (3) it is simple to implement [122].

#### 5.4.9. Naïve Bayes

The Naïve Bayes classifier is a probabilistic classifier based on applying the Bayes theorem under strong independence assumptions between features [123]. It is considered a supervised learner. A query image is represented by a set of features which are assumed to be independently sampled from a class-specific feature space. Then a kernel density estimation allows the Bayesian network models to achieve higher accuracy levels [123,124]. The Naïve Bayes Classifier can produce very accurate classification results with a minimum training time in comparison with conventional supervised or unsupervised methods.

#### 5.4.10. Decision Trees

Decision trees use tree-like models of decisions and their possible effects producing human-readable rules for the classification task [125]. Decision trees take the form of yes or no questions and therefore they are easily interpreted by people. The learning algorithm applies a rapid search for the many possible combinations of decision points to find the points that will give the simplest tree with the most accurate results. When the algorithm is run, one sets the maximal number of decision points, i.e., the depth, and the maximal breadth to be searched. At the end the algorithm determines how many decision points are required to achieve better accuracy. A decision tree model has high variance and low bias which leads to unstable output, and very sensitive to noise.

#### 5.4.11. Other Machine Learning Methods

New approaches such as federated learning, interactive reporting, and synoptic reporting may help to address data availability problem in the future; however, curating and annotating data, as well as computational requirements, remain substantial barriers to machine learning applications for MRI data [126].

### 5.5. Which ML Method Is Best for Identifying Diagnostic MRI Biomarkers

The best ML methods applied for MRI data analysis should be able to learn useful semantic features from MRI imaging data and lead to improved models for performing medical diagnosis tasks efficiently [122]. However, training good ML models requires large amount of labelled data that may not be available; it is often difficult to obtain a sufficient number of labelled images for training models. In many scenarios the dataset in question consists of more unlabeled images than labelled ones. Therefore, boosting the performance of ML models by using unlabeled as well as labelled data is an important but challenging problem [122].

Many ML methods, particularly deep learning, has boosted medical image analysis for disease diagnosis over the past years. Around 2009, it was realized that deep artificial neural networks (DNNs) were outperforming other established modeling methods on a number of important benchmarks [65]. Currently, deep neural networks are considered the state-of-the-art machine learning models across a variety of areas, from MRI image analysis to natural language processing, and widely deployed in academia and industry [103]. However, there are many challenges for the introduction of deep learning in clinical settings. Challenges are related to data privacy, difficulties in model interpretability and workflow integration.

Despite the large number of retrospective studies (Figure 2), there are fewer applications of deep learning in the clinic on a routine basis [127]. The three major use cases that deep learning can have in MRI diagnostics: (1) model-free image synthesis, (2) model-based image reconstruction, and (3) image or pixel-level classification [127]. Hence, deep learning has the potential to improve every step of the MRI diagnostic workflow and to provide value for every user, from the technologists performing the scan, the physicians ordering the imaging, the radiologists providing the interpretation, and most importantly, the patients who are receiving health care.

### 5.6. Assessment of Model Performance

For classification models, model performance is usually assessed by generating a confusion matrix and calculating several statistics indicative of model accuracy. In the case when MRI images belong to two classes (e.g., cancer and non-cancer), a 2 × 2 confusion matrix can be defined, where N_(1)_ and N_(0)_ are the numbers of MRI images in the data set that belong to classes (1) and (0), respectively. TP, TN, FP, and FN are the number of true positives (malignant MRI predicted as malignant MRI), true negatives (benign MRI predicted as benign MRI), false positives (benign MRI predicted as malignant MRI), and false negatives (malignant MRI predicted as benign MRI), respectively. The following classification accuracy characteristics associated with confusion matrices are widely used in classification machine learning studies: the true positive rate (TPR) also known as recall (R) or sensitivity (SE = TP/N_(1)_), specificity (SP = TN/N_(0)_), the false positive rate (FPR) which is 1-specificity, precision (*p* = TP/TP + FP) and enrichment *E* = (TP)N/[(TP + FP)N_(1)_]. Normalized confusion matrices can be also obtained from the non-normalized confusion matrices by dividing the first column by N_(1)_ and the second column by N_(0)_. Normalized enrichment can be defined in the same way as *E* but is calculated using a normalized confusion matrix: *E*_n_ = (2TP)N_(0)_/[(TP)N_(0)_ + (FP)N_(1)_]. *E*_n_ takes values within the interval of [0, 2] [98,128].

The receiver operating characteristic (ROC) curve is then created by plotting the TPR against the FPR at various thresholds. ROC and precision-recall (PR) analyses are usually performed side by side, and the area under the curve (AUC) is calculated to assess model performance in each case [129]. Both ROC-AUC area under the curve of receiver operating characteristic curves and PR-AUC area under the curve of precision-recall curves are widely used to assess the performance of ML methods for MRI biomarkers [100,129,130].

However, other model performance metrics have been calculated for imbalanced datasets that are usually encountered in the classification datasets. One of these metrics is the correct classification rate CCR which has been suggested as a better measure of model accuracy [98,99], using the equation below:

CCR=0.5

where and are the number of correctly classified and total number of compounds of class *j* (*j* = 1, 2).

The accuracy of MRI biomarkers for benign/malignant discrimination has improved dramatically approaching values higher than 90%; and a performance exceeding 80% classification sensitivity and specificity [19,37,131,132,133].

## 6. Types of MRI Biomarkers According to Clinical Use

### 6.1. Diagnostic Biomarkers

The Prostate Imaging Reporting and Data System (PI-RADS) has been approved as a diagnostic biomarker in prostate cancer employing multiparametric MRIc [134]. Additionally, the PROMIS study [135,136] has emphasized the contribution of multiparametric MRI in the examination of prostate cancer patients. In this study, 740 male patients were enrolled, 576 men experienced multiparametric MRI followed by template prostate mapping and transrectal ultrasound (TRUS) biopsy [135,136]. Results showed that multiparametric MRI is more sensitive than (93%, 95% confidence interval (CI) 88–96%) TRUS biopsy (48%, 42–55%, *p* < 0.0001) [135,136]. Risk grades evaluate the probability of clinically approved cancer; PI-RADS 5 very high, PI-RADS 4 high, PI-RADS 3 intermediate, PI-RADS 2 low, and PI-RADS 1 very low [1]. A meta-analysis procedure has identified sensitivity (0.74) and specificity (0.88) for prostate cancer with PI-RADS [137,138].

### 6.2. Prognostic Biomarkers

Prognostic imaging biomarkers are used for cancer staging in order to divide patients into different risk groups [1]. MRI is considered the basic staging probe for diverse cancers such as rectal cancer [1]. The TNM stage indicates inclusive survival out of 5 years; stage I (localized, T1/2), node negative: 95% compared to stage IV (metastatic, any T or N: 11%). MRI reflects a predictive role including patellofemoral syndrome (PFS) and resection margin [139,140,141].

### 6.3. Response Biomarkers

Response biomarkers evaluate the tumor’s response to treatment which is classified into four classes: progressive disease, stable disease, partial response, complete response. This classification depends on the size of modification for particular lesions which are >1 cm, or nodes which are >1.5 cm axis (Table 3) [1]. The RECIST protocol offers a structured and comprehensive measurement of response to treatment in clinical studies [32]. RECIST is significant response biomarker in clinical studies and is employed as a surrogate marker [1].

## 7. Types of MRI Biomarkers Based on Quantitative Ability

### 7.1. Semi-Quantitative Recording Systems

The output of semi-quantitative scores are extensively recruited because visual diagnosis is appropriate and related to scoring output [5]. The MRI recording systems for hypoxic-ischemic encephalopathy (HIE) in neonates by T1-weighted (W), T2-W, and diffusion-W images demonstrated higher post-natal scores accompanied with inadequate brain functions [142]. Similarly, high T2-W scoring of cervical spondylosis was linked to illness status and implications [143,144]. Imaging of osteoarthritis is significant for diagnosis process [145]. Internet-based knowledge transfer methods employing the well-established recording protocols showed harmony between imaging and medical specialty in explaining T2-W outcome [146]. Identical recording has been used in multiple sclerosis [147] and rectal wall diagnosis [148]. ^18^Fluoro-2-deoxy-D-glucose (^18^FDG) positron emission tomography–computed tomography (PET-CT) imaging has been applied in lymphoma evaluation [149]. Similar scoring has been used in breast, prostate, liver, thyroid, and bladder imaging cancers [150,151,152,153]. MRI scoring has been applied for identifying gynecological malignancies [154] and scoring of renal cancer [155]. Physical evaluation of lung nodule diameter and volume doubling time (VDT) has been widely used in diagnosis, identifying, screening, and response anticipating [156,157].

### 7.2. Quantitative Recording Systems

Quantitative assessment has been frequently used in size and/or volume measurement. Size contributes in measuring benign and malignant diseases [158]. Measuring of ventricular size on ECG is versatile and linked to medical protocol [158,159]. Left ventricular ejection fraction has been assessed by ultrasound and MRI. Rheumatoid arthritis with aberrant bone features has been recorded with CT as an indicator of the illness progress [160]. RECIST (1.0 and 1.1) [158] assesses cancer prognosis; RECIST measurements are simple, but ambiguous and not reliable [161,162]. The fact that diverse studies have related volume to disease diagnosis [163,164,165,166], volume has not been authenticated in clinical records due to the requirement of splitting of abnormal shaped cancers. Volume is a surrogate for disease progress and response [167]. The metabolic tumor volume (MTV) measuring by PET has been related to survival [168,169]. Furthermore, MTV is an indicator of lymphoma and is considered a biomarker for treatment response [170,171,172]. Eventually, the presence of automated volume partitioning is crucial for treatment approval [5].

### 7.3. Quantitative Imaging Biomarkers

Quantitative imaging biomarkers that delineate tissue hallmarks such as hypoxia, fibrosis, necrosis, perfusion, and diffusion elaborate the illness state and express histopathology [5]. Numerous quantitative hallmarks can be integrated into mathematical equations to evaluate disease progress and changes during time intervals [5]. Organization of physiological databases is elaborated based on disease existence and type accompanied with scoring according to clinical data to extract anticipative models that serve as diagnosis-support tools. Such model has been provided for brain data inquiring approved and well-organized databases [173]. Exploiting quantitative data embedded in images along with demanding protocols for accession and scoring linked with machine learning algorithms have been applied in neurodegenerative disease and treatment protocol [174,175].

## 8. Radiomic Signature Biomarkers

Radiomics elaborates the extraction and measurement of quantitative features from radiographic images [24,176]. Radiomics expresses abnormal physiological testing related with other “omics” like proteomics, metabolomics, and genomics [177]. Numerous radiomic hallmarks can be derived from a region or volume of interest (ROI/VOI), calculated manually, semi-automatically, or automatically by computational mathematical algorithms [5]. The summary of all hallmarks is the radiomics signature that is distinct for a tissue, patient, patient group, or disease [85,178]. Radiomics signature depends on imaging information type (PET, MRI, CT), image parameter and implementation, machine-learning, and VOI/ROI segmentation [179].

Though radiomic shot is diverse and not tissue selective, it identifies treatment prognosis, resistance, and survival [180]. Radiomics assist in decision making for treatment protocol and risk prioritization [5]. Interestingly, X-ray mammography, CT, MRI, PET, and single-photon emission computed tomography (SPECT) demonstrated potential results resulting in interpretation benign disease [181]. Improving of image property and data regulation is obligatory for expansive usage. Radiomic fingerprints are multi-component data and records for computational strategies such as neural networks Furthermore, reliability of signatures derived from CT and MRI data is adequate [182,183].

## 9. MRI Biomarker Standardization

The reproducibility of radiomic studies remains a non-trivial challenge for prioritizing MRI biomarkers. The lack of standardized definitions of radiomics features has resulted in studies that are difficult to reproduce and validate [184]. Additionally, inadequate reporting by these studies has impeded reproducibility further. As a result, the Image Biomarker Standardization Initiative (IBSI) was established to address these challenges by fulfilling the following objectives: “(a) establish nomenclature and definitions for commonly used radiomics features; (b) establish a general radiomics image processing scheme for calculation of features from imaging; (c) provide data sets and associated reference values for verification and calibration of software implementations for image processing and feature computation; and (d) provide a set of reporting guidelines for studies involving radiomic analyses” [184]. Additionally, the methodologic quality of radiomic studies to produce stable features that can be linked to cancer biology can be evaluated using the radiomics quality scoring (RQS) [185].

In order to address the problem of inadequate reporting, the American College of Radiology (ACR) endorsed a Reporting and Data Systems (RADS) framework which provides standardized imaging terminology and report organization to document the findings imaging procedures [2,4]. Additionally, modern picture archiving and communication systems (PACS) [186] possess digital modalities which are connected via the digital imaging and communications in medicine (DICOM) protocol [187]. The DICOM header usually provides information to interpret the body part examined and patient attributes such as position. The type of reported information can be adjusted from the machine settings before performing the imaging procedure.

## 10. Selected Examples on MRI Biomarkers in Solid Tumors

### 10.1. MRI Biomarkers for Prostate Cancer

Prostate cancer (PCa) is one of the most prevalent cancers occurring in men. The early detection of PCa is essential for successful treatment and to increase survival rate [188]. Lately, magnetic resonance imaging (MRI), has gained a progressively significant role in the diagnosis and early detection of PCa [189]. Multiparametric MRI (mpMRI) has been proven as a valuable procedure in detection, localization, risk stratification and staging of clinically significant prostate cancer (csPCa). Multiparametric MRI is based on combining the morphological evaluation of T2-weighted imaging (T2WI) with diffusion-weighted imaging (DWI), dynamic contrast-enhanced (DCE) perfusion imaging and spectroscopic imaging (MRSI) to better assess prostate morphology and identify tumor growth [190,191,192,193,194,195].

In addition, mpMRI-targeted biopsies have been shown to provide more accurate diagnosis of csPCa and to reduce the number of repeated biopsies needed for correct diagnosis relative to the transrectal ultrasound-guided biopsies [196]. However, mpMRI still suffers from inter-personnel agreement and variability of diagnostic accuracy based on the specialist’s experience [29,190,197,198,199].

Numerous studies in the literature described the potential role of employing MRI and ML for the analysis of prostate gland tissues and cellular densities to detect PCa. For example, McGarry et al. [200] established an adequate model to obtain a stable fit for ML MRI detection of augmented epithelium and diminished lumen density areas asserting high-grade PCa.

In addition, the volumetric regions of interest (ROI) analysis of index lesions on mpMRI [201] that is based on data available from T2-weighted, DWI and DCE images in combination with a support vector machine (SVM) ML, has been shown to significantly increase he diagnostic performance of PI-RADS v2 in clinically relevant prostate cancer.

Another useful application of ML MRI has been reported for the accurate distinction of stromal benign prostatic hyperplasia from PCa in the transition zone, a challenging diagnosis particularly in the presence of small lesions. Using ML based statistical analysis of quantitative features such as ADC maps, shape, and image texture, immense diagnostic accuracy in the of differentiation between small neoplastic lesions from benign ones was demonstrated [202].

The implication and feasibility of multiparametric machine learning and radiomics have been frequently discussed in literature for the identification and segmentation of clinically significant prostate cancer [203]. A deep learning–based computer-aided diagnostic approach for the identification and segmentation of clinically significant prostate cancer in low-risk patients was recently reported by Arif et al. [204]. The average sensitivity was 82–92% at an average specificity of 43–76% with an area under the curve (AUC) of 0.65 to 0.89 for several lesion volumes ranging from >0.03 to >0.5 cc. In addition, supervised ML classifiers have been used to successfully predict clinically significant cancer prostate cancer utilizing a group of quantitative image-features and comparing them with conventional PI-RADS v2 assessment scores [205].

### 10.2. MRI Biomarkers for Brain Tumors

Brain tumors are graded to benign (grade I and II) and malignant tumors (grade III and IV). Non-progressive (benign tumors) are originated in the brain but grow slowly and tend not to metastasize to other parts of the body while the malignant tumors grow rapidly with poor differentiation. They maybe originated in the brain and metastasize to other organs (primary) or initiated elsewhere in the body and migrated to the brain (secondary tumor) [206,207].

Magnetic resonance imaging (MRI) is a universal method for differential diagnosis of brain tumors. However, imaging with MRI is always susceptible to human subjectivity and early brain–tumor detection usually depends on the expertise of the radiologist [208], thus accurate diagnosis requires additional medical procedures such as brain biopsy. Unfortunately, biopsy of the brain tumor requires major brain surgery that puts patients at risk. The advancement of new technologies, such as machine learning has had substantial impact on the use of MRI as diagnostic tool for brain tumors. In addition, imaging biomarkers are routinely used for prognosis, and following up on treatment approaches for brain tumors.

Cheng et al., developed databases to classify tumor types using augmented tumor region of interest, image dilatation, and ring-form partition. Intensity histogram and gray level co-occurrence matrix were used to extract features and achieve an accuracy of 91.28% [209]. Additionally, the convolutional neural network (CNN) has made enormous improvement in the field of image processing, with particular impact on segmentation and classification of brain tumors. Brain tumor segmentation methods can be generally classified into three groups: based on traditional image algorithms, based on machine learning, and based on deep learning. Therefore, the segmentation method based on the CNN is widely used in segmentation of lung nodules, retinal segmentation, liver cancer segmentation, and glioma segmentation [210]. Milica et al. [211] recently reported a new CNN architecture for brain tumor identification, with good generalization capability and good execution speed, that was tested on T1-weighted contrast-enhanced magnetic resonance images.

The use of machine learning and radiomics have been suggested for various ap-plications in the imaging and diagnosis of meningiomas with promising outcomes [212]. Differentiating between meningeal-based and intra-axial lesions using MRI can be challenging in some cases. Banzato et al. [213] reported the use of CNN to extract and analyze complex sets of data to discriminate between meningiomas and gliomas in pre- and post-contrast T1 images and T2 images. In their study, an image classifier combining CNN and MRI, was developed to distinguish between meningioma and glioma lesions with accuracy of 94% (MCC = 0.88) on post-contrast T1 images, 91% (MCC = 0.81) on pre-contrast T1-images and 90% (MCC = 0.8) on T2 images.

## 11. Assigning and Interpreting of Proper Imaging Biomarkers to Confirm Decision-Making

Computerized quantitative evaluations are convenient to implement in machine learning systems. Therefore, the limit values, that determine the possibility of disease occurrence compared to no disease, should be recognized [214]. Such recognized values potentiate the use of imaging a computational biopsy. Assignment of biomarker selection depends on treatment protocol and disease response. Non-selective treatment, tissue necrosis is considered; therefore, biomarkers that evaluate increased free water (CT Hounsfield units) or decreased cell density (ADC) are beneficial. However, selective-treatment such as anti-angiogenesis therapy, perfusion measurements (CT, MRI, and US) as selective biomarkers are considered [215]. Non-selective and selective agents terminate cancer metabolism; therefore, in glycolytic cancers fluorodeoxyglucose (FDG) assessments are reliable [216]. The deformity of tissues after surgery or changes in normal tissues after radiotherapy [217] as well as decrease in quantitative variations between metastatic and non-metastatic tissue [218] should be considered.

## 12. Progress in Quantitative Imaging Biomarkers as Decision-Making Tools in Clinical Practice

Biomarkers should be reliable, reproducible, in addition to being biologically, clinically and cost effective [18]. While reproducibility is a necessity, it is not frequently observed in practice [219] because incorporating of fundamental research in clinical studies is an arduous task for both patients and investigators. Technical verification determines whether a biomarker can be reproduced in different places on diverse panels. Technical validation may take place after biological validation especially for biological changes that modify imaging biomarker traces that endorse the values assigned to biomarkers. Correlation between clinical and technical validation precedes the assignment of biomarker for specific use. The implementation of imaging biomarkers in clinical diagnosis is assessed as a parameter in medical management such as circulating cancer DNA is specific for cancer identification. The incorporation of imaging biomarkers such as tissue and liquid biomarkers replaces old and simple protocols. The robustness of biomarker’s cost is significant in economically limited medical systems [220]. Further imaging protocols are expensive in contrast to liquid-and tissue-derived biomarkers. Health financial measurement is beneficial for incorporating a new biomarker in clinical diagnosis. The use of imaging biomarkers is a key tool in supporting medical diagnosis protocols.

## 13. The Challenges for Prioritizing MRI Biomarkers

Despite major advancements in big data analysis and machine learning methods, the development of quantitative imaging biomarkers that can be exploited effectively in medical decisions is hampered by major challenges related to data availability, variability and lack of reliability [3]. Data availability is impacted by limitations related to data sharing, data ownership and patient privacy [221]. Furthermore, the absence of international standard protocols along with quality assurance (QA) and quality control (QC) procedures contributes in an inadequate quantification and interpretation of MRI biomarkers [4,18,222]. This prevents physicians from extracting the required clues for interpreting disease status [223], or for assessing the efficacy of treatment protocols [22]. Additionally, it decreases our capability of merging MRI biomarkers that have been extracted from different imaging methods [1].

## 14. Conclusions

In this article, we have provided an overview of ML and MRI data. We discussed the nature of MRI data, local and global features, and most frequently used ML methods for model building to prioritize MRI biomarkers. These biomarkers have the potential to revolutionize cancer care, providing a platform for personalized, high-quality, and cost-effective health care for oncology patients. The application of ML methods for the analysis of MRI data has led to the development of disease-specific biomarkers for many cancers including hematological, lymphatic and solid tumors. Neural networks, contrastive learning and deep learning are becoming the leading methods for prioritizing MRI biomarkers. The performance of MRI biomarkers is now exceeding 80% for most methods and cancer types. MRI biomarker performance for disease classification (i.e., malignancy vs. benign) is exceeding 90% for deep learning, neural networks and SVM. Advances in deep learning and AI are expected to revolutionize MRI biomarkers and increase their utility for preclinical and clinical applications in oncology.

## Figures and Tables

**Table 1:** Imaging biomarkers for disease detection with examples.

Disease	Biomarker	Quantitative (Q)/Semi-Quantitative (SQ)/Non-Quantitative (NQ)	Biomarkers Uses
Malignant disease	Lung RADS, pancreatic cancer action network (PanCan), national comprehensive cancer network (NCCN) criteria [25,26]	SQ	AUC for malignancy 0.81–0.87 [27]
	CT blood flow, perfusion, permeability measurements	Q	Sensitivity 0.73, specificity 0.70 [28] AUC 0.75, sensitivity 0.79, specificity 0.75 [29]
	Breast imaging (BI)-RADS [30] Prostate imaging (PI)-RADS [29] Liver imaging (LI)-RADS [31]	SQ	positive predictive value (PPV) BI-RADS 0 14.1%, BI-RADS 4 39.1%, BI-RADS 5 92.9% PI-RADS 2 pooled sensitivity 0.85, pooled specificity 0.71 pooled sensitivity for malignancy 0.93
	Apparent diffusion coefficient (ADC)	Q	Liver AUC 0.82–0.95 Prostate AUC 0.84
	RECIST/morphological volume	Q	Ongoing guidelines for treatment evaluation [32]
	Positron emission response criteria in solid tumors (PERCIST) /metabolic Volume [33]	Q	Ongoing guidelines for treatment evaluation [32]
Liver cancer Recurrent glioblastoma	Dynamic contrast enhanced (DCE) metrics (perfusion parameters K^trans^, K_ep_, blood flow, Ve)	Q	Hepatocellular cancer AUC 0.85, sensitivity 0.85, specificity 0.81 [29] Brain- K^trans^ accuracy 86% [34]
Cancer Sarcoma [35] Lung cancer [36]	^18^FDG- standardized uptake value (SUV)	Q	Sarcoma-sensitivity 0.91, specificity 0.85, accuracy 0.88 Lung-sensitivity 0.68–0.95
Cancer	Targeted radionuclides [37] In-octreotide [38,39] ^68^Gallium (Ga)-DOTA-TOC [39] and ^68^Ga DOTA-TATE [39,40,41] ^68^Ga prostate-specific membrane antigen (PSMA) [42]	NQ	Sensitivity 97%, specificity 92% for octreotide [43] Sensitivity 100%, specificity 100% for PSMA [44]
Brain cancer	Dynamic susceptibility contrast (DSC)-MRI	SQ	AUC = 0.77 for classifying glioma grades II and III [45]
Glioma	Adjuvant paclitaxel and trastuzumab (APT) trial	Q	APT accords with cancer grade and Ki67 index [46]
Rectal cancer Lung cancer	DCE-CT parameters Blood flow, permeability	Q	Blood flow 75% accuracy for detecting rectal cancers with lymph node metastases [47] CT permeability anticipated survival regardless of treatment in lung cancer [48]
Cervix cancer Endometrial cancer Rectal cancer Breast cancer	DCE-MRI parameters	Q	Cancer volume with increasing metrics is considered a significant independent factor for disease-free survival (DFS) and overall survival (OS) in cervical cancer [49] Low cancer blood flow and low rate constant for contrast agent intravasation (kep) correlated with high risk of endometrial cancer [50] K^trans^, K_ep_ and Ve are higher in rectal cancers accompanied with metastasis [51] K^trans^, iAUC qualitative and ADC anticiptate low-risk breast cancers (AUC of combined parameters 0.78)
Diverse cancer types [52,53]	Radiomic signature [54] DCE-MR parameters	Q	Data endpoints, feature detection protocols, and classifiers are important factors in lung cancer prediction [55] Radiomic signature is significantly associated with lymph node (LN) status in colorectal cancer [56] Evaluating therapeutic effect subsequent to antiangiogenic agents [57]
Lymphoma	Deauville or response evaluation criteria in lymphoma (*RECIL*) score on ^18^F-FDG-PET	SQ	Assessment of lymphoma treatment in clinical trials employs the summation of longest diameters of three target lesions [58]
Breast cancer [59] Prostate cancer [60]	Receptor tyrosine-protein kinase erbB-2, CD340, and HER2 prostate-specific membrane antigen (PSMA)	SQ	Selective cancer receptor; investigation of cancer treatment on receptor expression. Assessing therapy response to antiangiogenic agents [57]
Oesophageal cancer	CT perfusion/blood flow	Q	Multivariate analysis detects blood flow as a predictor of response [61]
Gastrointestinal stromal cancers	CT density HU	Q	Decrease in cancer density of > 15% on CT associated with a sensitivity of 97% and a specificity of 100% in identifying PET responders compared to 52% and 100% by RECIST [61]

**Table 2:** A comparison between popular machine learning algorithms used for the prioritization of diagnostic MRI biomarkers [88,100,101,102].

ML Method	Diagnostic Characteristics
Artificial Neural Network (ANN)	The mathematics behind the classification algorithm is simple. The non-linearities and weights allow the neural network (NN) to solve complex problems. Long training time is required for numerous iterations over the training data. Tendency for overfitting. Numerous additional tuning hyperparameters including # of hidden layers/hidden nodes are required for determining optimal performance.
Contrastive Learning	Self-supervised, task-independent deep learning technique that allows a model to learn about data, even without labels. Learns the general features of a dataset by teaching the model which data points are similar or different. Can potentially surpass supervised methods. May yield suboptimal performance on downstream tasks if the wrong transformation invariances are presumed.
Decision Trees (DTs)	Easy to visualize Easy to understand. Feature selection plays a dominant role in the accuracy of the algorithm. One set of features can provide drastically different performance than a different set of features, therefore, large Random Forests can be used to alleviate this problem. Prone to overfitting.
Deep Learning (DL)	Can perform both image analysis (deep feature extraction) and construction of a prediction algorithm, eliminating the need for separate steps of extracting radiomic features and using that that to train a prediction model. Can learn from complex datasets and achieve high performance without requiring prior feature extraction. Permits massive parallel computations using GPUs. Requires additional hyper-parameters tune the model for better performance including the number of convolution filters, the size of the filters, and parameters involved in the pooling. Requires large training sets and it is not an optimal approach for pilot studies or internal data with small datasets. Computationally-expensive.
*k* Nearest Neighbor (*k*NN)	Easy to implement as it only requires the calculation of the distance between different points on the basis of data of different features. Computationally-expensive for large datasets. Does not work well with high dimensionality as this will complicate the distance calculating process to calculate distance for each dimension. Sensitive to noisy and missing data. Requires feature scaling. Prone to overfitting.
Logistic Regression	Constructs linear boundaries, i.e., it assumes linearity between dependent and independent variables. However, linearly separable data is rarely found in real-world scenarios.
Naïve Bayes	Models are faster to train and are simple, datasets and inferior performance on larger datasets. The Naïve Bayes classifier has generally shown to have superior performance in comparison to the Logistic Regression classifier on smaller datasets. Less potential for overfitting. Shows difficulties with complex datasets due to being linear classifiers.
Random Forests (RFs)	Less prone to overfitting, and it reduces overfitting in decision trees and helps to improve the accuracy. Outputs the importance of features which is a very useful for model interpretation. Works well with both categorical and continuous values, for both classification and regression problems. Tolerates missing values in the data by automating missing value interpretation. Output changes significantly with small changes in data.
Self-supervised Learning (SSL)	Suitable for large unlabeled datasets, but its utility on small datasets is unknown. Reduces the relative error rate of few-shot meta-learners, even when the datasets are small and only utilizing images within the datasets.
Support Vector Machines (SVM)	Simple mathematics are behind the decision boundary Can be applied in higher dimensions. Time-consuming for large datasets, especially for datasets with larger margin decision boundary. Prone to overfitting. Sensitive to noisy and large datasets.

**Table 3:** Response categories according to changes in tumor lesions.

Category	RECIST	
	Target Lesions	Nontarget Lesions
Progressive disease (PD)	>20% ↑ in the sum of target lesions (TL) diameters. Absolute ↑ (5 mm). Appearance of new lesions.	Clear progress of surviving nontarget lesion. Appearance of new lesions.
Stable disease (SD)	Neither PD nor PR	Continuity of ≥ 1 nontarget lesion
Partial response (PR)	>30% ↓ in the sum of TL	Non-PD/CR
Complete response (CR)	Disappearance of TL. All nodes < 10 mm Non-pathological nodes	Disappearance of nontarget lesions. All nodes < 10 mm Non-pathological nodes

## Data Availability

Data is contained within the article.
